# The impact of the COVID-19 pandemic on adverse fetal outcomes: A cross-sectional study

**DOI:** 10.1097/MD.0000000000033887

**Published:** 2023-05-26

**Authors:** Andrea Dagelić, Emma Mulic, Ivana Kuzmic Prusac, Sandra Zekic Tomas

**Affiliations:** a Department of Obstetrics and Gynecology, University Hospital Centre Split, Spinciceva 1, Split, Croatia; b Department of Internal Medicine, North Älvsborgs County Hospital, Lärketorpsvägen, Trollhättan, Sweden; c Department of Pathology, University Hospital Centre Split, Spinciceva 1, Split, Croatia.

**Keywords:** COVID-19, fetal outcomes, healthcare system, pandemic

## Abstract

The coronavirus disease 2019 (COVID-19) pandemic has been one of the most damaging pandemics in all of human history. Some of the most vulnerable groups within society such as pregnant women and children have also been affected. This observational research, cross-sectional study was conducted to investigate if there was any difference in the incidence of unfavorable outcomes in pregnancy such as miscarriage, intrauterine fetal demise, and early neonatal death during the year prior to the pandemic and the year of the COVID-19 pandemic. This retrospective study was conducted at the University Hospital of Split at the Department of Pathology, Forensic and Cytology and Department of Obstetrics and Gynecology of the same hospital. All data was collected in the time period from March 1st, 2019, to March 1st, 2021. The study included all pregnant women who had an unfavorable pregnancy outcome such as miscarriage and intrauterine fetal demise, as well as early neonatal death at the University Hospital of Split within the time frame mentioned previously. There was no statistically significant difference in the incidence of adverse pregnancy outcomes in the year prior to the pandemic and during the year of the COVID-19 pandemic. Our study showed that the pandemic did not have a negative effect on pregnant women and their fetuses; there was no increase in miscarriage, intrauterine fetal demise, or perinatal death during the year of the pandemic.

## 1. Introduction

Coronavirus disease 2019 (COVID-19), which is caused by severe acute respiratory syndrome coronavirus-2 (SARS-CoV-2), has caused one of the most damaging pandemics in recorded human history.^[1]^ It was first discovered in Wuhan, China in December 2019 and it started spreading widely and rapidly. On March 11th 2020, the World Health Organization declared COVID-19 a global pandemic.^[2]^ By May 22nd 2022, there were more than 518 million cases reported worldwide and over 6 million deaths had been confirmed.^[3]^ Since the initial outbreak of the COVID-19 pandemic, many questions have arisen in regards to the effect of COVID-19 on pregnant women, specifically relating to whether there is an increased susceptibility to the SARS-CoV-2 infection and whether there is an increased risk for severe diseases. Furthermore, the question of whether it could result in any unfavorable outcomes during pregnancy arose. The risk of vertical transmission of the virus from the mother-to the fetus has also been of great concern.^[4,5]^ The clinical manifestations of COVID-19 in pregnant women are similar to those of nonpregnant women.^[6]^ The rate of clinical complications of COVID-19 infection in pregnant women is very similar to the general population.^[7]^ A cohort study conducted by the World Health Organization, which included a sample of 147 COVID-19 positive pregnant women, reported that 8% became severely ill and only 1% became critically ill. This data suggested that pregnant women with the COVID-19 infection had milder clinical manifestations.^[6]^ During the initial phase of the COVID-19 pandemic, researchers could not find a difference significant enough when comparing women infected by the COVID-19 virus to healthy women in regards to the frequency of fetal distress, preterm labor and neonatal asphyxia.^[8]^ However, mounting evidence that SARS-CoV-2 has an adverse outcome on pregnancies are beginning to appear, whilst vertical transmission from mother-to-fetus rarely appears. It has now been reported that pregnant women with confirmed SARS-CoV-2 infection have an increased risk of preterm birth, preeclampsia, and stillbirth.^[5]^ Spontaneous abortion, also referred to as miscarriage, is another unfavorable outcome that has been associated with SARS-CoV-2 infected pregnant women.^[9,10]^ This is believed to be due to the changes and inflammation of the placenta leading to fetal growth retardation and then consequently inducing spontaneous abortion.^[11]^ An infection with SARS-CoV-2 during the preconception period and during the first half of pregnancy has been associated with an increased risk for both miscarriage and failure of embryo implantation.^[12]^ Pregnancy outcomes are not only influenced by the direct impact of COVID-19, but also by disruptions to the healthcare system caused by the pandemic. These disruptions have contributed to adverse effects in all pregnant women, including those who were not infected with SARS-CoV-2. A global systematic review observed an increased rate of stillbirth, a decline in maternal mental health, and an increase in the rate of ectopic pregnancy rupture representing a delay in the care system during the pandemic compared to the one in the years prior to the pandemic.^[13]^ The aim of this study was to investigate if there was any difference in the incidence of adverse fetal outcomes during the year prior to the pandemic and the year of the COVID-19 pandemic in The University Hospital Split.

## 2. Methods

### 2.1. Study design

This cross-sectional retrospective study included all pregnant women who had an unfavorable pregnancy outcome such as miscarriage and intrauterine fetal demise, as well as an early neonatal death at the University Hospital of Split. All data studied were collected from Department of Pathology and Department of Obstetrics and Gynecology at The University Hospital Split in the time period from March 1st 2019 to March 1st 2021. The collection of data for this study was extracted from the department of Pathology, Forensic and Cytology as well as the Department of Gynecology and Obstetrics at the University Hospital of Split.

Inclusion criteria were as follows: complete and incomplete miscarriages, intrauterine fetal demise and early neonatal death in the time period mentioned previously. Early neonatal death included neonatal death within 7 days of delivery. Exclusion criteria were the cases with incomplete medical records and induced abortions for any reason.

The study recorded the following data: maternal age, gestational age, and medical history regarding previous pregnancies for the miscarriage samples. For the intrauterine fetal demise and early neonatal death gender of the fetus, gestational age in weeks, age of neonate if early neonatal death was in order, as well as death cause were recorded, together with general information regarding the mothers.

After obtaining the data, it was processed using Microsoft Excel to create tables and perform additional analysis. ReCoDe classification was used to classify cause of death for intrauterine fetal demise and early neonatal death.^[14–16]^

### 2.2. Statistical analysis

The statistical analysis of the data was performed using Med Calc software (Med Calc software, Ostend, Belgium). Data distribution was estimated using the Kolmogorov-Smirnov test. Various tools were used to interpret the statistical significance of data. The t-test was used for the normal distribution of data, while the Mann–Whitney *U* test was used for data without the normal distribution. χ2-square test (also called chi-squared test) was used to estimate the qualitative variables among the different groups. Data is presented as an arithmetic mean with standard deviation or as a median with minimum and maximum values. The statistically significant value was set at *P*˂.05.

## 3. Results

This study investigated adverse pregnancy outcomes. The period of March 1st, 2019 to February 29th, 2020 was designated as the “group before the COVID-19 pandemic” while the period from March 1st, 2020 to March 1st, 2021 was designated as the “group during the COVID-19 pandemic”. Unfavorable outcomes of pregnancies were further divided into miscarriages/abortions and loss of pregnancies that occurred after the 21st gestational week and onwards, including early neonatal death. There were 4293 births in the year before the COVID-19 pandemic and 4153 births in the year during pandemic. Throughout the study period, a total of 691 miscarriages were recorded, of which 392 (56.7%) occurred during the year before the COVID-19 pandemic, while 299 (43.3%) occurred during the year of the COVID-19 pandemic. The rest of the demographic data are presented in Table 1. All patients who experienced a miscarriage or stillbirth were tested for the COVID-19 infection. Only 2 pregnant women who had a miscarriage during COVID-19 tested positive for SARS-CoV-2 infection at the time of the miscarriage. All patients who had a stillbirth tested negative for the SARS-CoV-2 infection at the time of stillbirth.

**Table 1:** Demographic data for the patients who had miscarriage in the year before and during COVID-19 infection.

	Before COVID-19 N = 392	During COVID-19 N = 299	*P* value
Mother’s age (yr)	33 (16–46)	34 (17–46)	.217*
Gestational week at the time of the miscarriage	9 (5–14)	9 (5–15)	.647†
Gravidity			<.001‡
0	82.6%	53.5%	
1	8.4%	20.1%	
2	5.1%	15%	
3	2%	8.7%	
4	1%	1.3%	
5	0.5%	0.3%	
6	0.3%	0.7%	
7	0	0	
8	0	0.3%	
Parity			<.001‡
0	85.2%	61.9%	
1	9.2%	19.4%	
2	3.6%	13.4%	
3	1%	4.3%	
4	0.5%	0.3%	
5	0.5%	0.7%	
Previous miscarriage/s	93.6%	83.6%	<.001‡
0	4.8%	9.7%	
1	0.8%	5.4%	
2	0.8%	1.3%	
3			
Habitual miscarriage	0.8%	1.3%	.267‡

COVID-19 = coronavirus disease 2019.

* Mann-Whitney U test;

† Student’s *t* test;

‡ Chi-square test

There was no statistically significant difference in the maternal age (z = 1.235; *P* = .217), gestational week at the time of miscarriage (t = 0.468; *P* = .647) and the frequency of habitual miscarriages (χ2 = 1.217; *P* = .267) between the 2 studied groups.

The majority of the patients that had a miscarriage in both groups didn’t have previous gravidity in their anamnesis. However, in the year during COVID-19, there was a statistically significant increase of patients who were pregnant at least 1 or 2 times prior to this miscarriage compared to the patients in the year before COVID-19 (χ2 = 47.683; *P* < .001). Likewise, patients who had a miscarriage during the year of COVID-19 had a higher frequency of previous parity and previous miscarriage compared to the patients who had a miscarriage in the year before the COVID-19 pandemic (χ2 = 54.004; *P* < .001) (χ2 = 21;174 *P* < .001).

Demographic data for the unwanted pregnancy outcomes after the 21st gestational week are presented in Table 2. There was no statistically significant difference between studied groups in regard to the gestational week (t = 0.859; *P* = .376), gender (χ2 = 0.000; *P* = .986), stillbirth (χ2 = 1.034; *P* = .309), and early or late neonatal death (χ2 = 0.268; *P* = .605). Causes of perinatal death were classified according to the ReCoDe classification system. There was no statistically significant difference between the studied groups as shown in Table 3 (χ2 = 5.426; *P* = .366). Likewise, there was no statistically significant difference between the studied groups in regards of the ReCoDe subclassification for every perinatal cause of death as follows: fetus category (χ2 = 5.741; *P* = .219), umbilical cord category (χ2 = 0.006; *P* = .936), placenta category (χ2 = 0.001; *P* = .975), amniotic fluid category (χ2 = 1.021; *P* = .312), and unclassified category (χ2 = 0.703; *P* = .402). In the umbilical cord category, namely in the subgroup other referred to hyper torsion of umbilical cord and causative fetal vascular malperfusion for both studied groups. Total of 15 perinatal deaths were classified as other in the placenta category, all of them had maternal vascular malperfusion.

**Table 2:** Demographic data for the fetal and neonatal death in regard of COVID-19 pandemic.

	Before COVID-19 N = 47	During COVID-19 N = 49	*P*
Gestational age (wk)	27 ± 6	26 ± 6	.376*
Gender M:F	25:22	27:22	.986†
Stillbirth	21 (45%)	28 (57%)	.309†
Neonatal death	Early 21 Late 5	Early 19 Late 2	.605†

COVID-19 = coronavirus disease 2019.

* Student’s *t* test.

† Chi-square test.

**Table 3:** Perinatal death classification according to ReCoDe classification and COVID-19 pandemic.

Categories	Before COVID-19	During	*P* value
	N = 47	COVID-19 N = 49	
Fetus	15 (32%)	10 (20%)	
Congenital anomalies	4	6	
Perinatal infection	7	2	
IUGR	1	0	.219*
Twin-Twin syndrome	1	2	
Nonimmune hydrops	2	0	
Umbilical cord	6 (13%)	7 (14%)	
Loop or Knot	0	1	.936*
Other	6	6	
Placenta	7 (15%)	12 (25%)	
Abruption	2	2	.975*
Other	5	10	
Amniotic fluid	18 (38%)	15 (31%)	.312*
Uterus	0	0	
Mother	0	1 (2%)	
Unclassified			
No relevant condition found	1 (2%)	4 (8%)	.402*
Insufficient information			

COVID-19 = coronavirus disease 2019, IUGR = intrauterine growth restriction.

* Chi-square test.

## 4. Discussion

At the beginning of the COVID-19 pandemic, medical doctors and researchers alike expressed concern that the COVID-19 infection would have a negative impact on pregnant women, and their unborn children. The whole health care system was overwhelmed resulting in reduced availability of medical staff for general population, pregnant women included. Likewise, pandemic itself brought negative impact on mental health worldwide, with pregnant women being equally affected. Taking this into consideration, our study aimed to investigate unfavorable pregnancy outcomes the year prior to the pandemic and to compare the results with those during the first year of pandemic.^[17]^

At the University Hospital of Split, all pregnant women who had undergone a medical abortion during the year of COVID-19 were tested for SARS-CoV-2 prior to the admission. However, only 2 patients tested positive at the time of miscarriage. The results of this study showed that out of the 691 miscarriages that occurred during the time period investigated, 392 (56.7%) of these occurred the year prior to the COVID-19 pandemic and 299 (43.3%) the year during the pandemic. The somewhat lower number of miscarriages during the year of COVID-19 pandemic as one of the key results may reflect the fact that the visits to the gynecologist were also reduced during this time. Women could have mistaken their early miscarriage with menstruation and thereby did not report it to their general practitioner or gynecologist. Additionally, during the COVID-19 pandemic the hospital admissions were limited, and resources were cut short, so, if possible, cases with uncomplicated miscarriage were handled by primary care gynecologists. The increased risk of COVID-19 transmissions in healthcare settings could have been another risk some pregnant women did not wish to take. Therefore, it is possible that some women had an early miscarriage without being aware of it, resulting in them not seeking medical care, thus leading to no medical report of their miscarriage.^[9,10,18,19]^ Rotshenker-Olshinka et al^[20]^ also reported that the COVID-19 pandemic environment does not seem to affect early first-trimester miscarriage rates.

Our study showed that in both investigated groups, the majority of patients who had a miscarriage had no prior gravidity in their obstetrical anamnesis. During the year of the COVID-19 pandemic there was a statistically significant increase of patient who had been pregnant at least once or twice prior to this miscarriage. These findings may be attributed to the fact that women who have had a previous pregnancy are more familiar with the various symptoms and signs that occur in early pregnancy, and as a result are more likely to seek medical attention if need be. This, in turn, increases the likelihood of the miscarriage being recorded in medical records and not going undocumented. The latter could also explain the fact that a higher frequency of previous parity and miscarriage was seen during the pandemic compared to the year prior.

There was no statically significant difference in gestational week at the time of miscarriage when comparing the group before the COVID-19 pandemic with the group during the COVID-19 pandemic. Also, there was no statistically significant difference in maternal age or frequency of habitual miscarriage. These results were expected due to the fact that the aforementioned data is usually constant and does not usually change frequently, especially with a smaller sample size and a short and limited time period.

A systematic review reported an increase rate of stillbirth in pregnant patients during the pandemic compared to that before the pandemic, and it was concluded that this was due to a delay in health care because of the COVID-19 pandemic.^[13]^ The above-mentioned study conflicts with a study conducted in the United States of America, which claimed that the rate of stillbirth before the COVID-19 pandemic closely resembled the incidence during the pandemic.^[21]^ Our study is in accordance with the aforementioned study as no statistically significant difference was seen in the studied groups regarding the incidence of stillbirth. Also, one of the key results was that no significant difference was seen in the incidence of early and late neonatal death. Siddiqui and Najam also reported that the rate of intrauterine fetal demise and neonatal death were less frequent during the pandemic.^[22]^ In conclusion, the study shows that the COVID-19 pandemic and its associated stressful and negative effects and the increased workload of healthcare staff did not have an effect on pregnant women seeking medical care at the University Hospital of Split. Despite the challenges that were brought by the pandemic, pregnant patients at the University Hospital of Split continued to receive appropriate health care and were not at a higher risk of experiencing stillbirths or early neonatal death as a result of the pandemic. Furthermore, stillbirth is defined as the termination of a pregnancy after the 20th week of gestation. Most expectant mothers are aware of their pregnancy and therefore attend routine medical checkups, which may contribute to a reduced incidence of stillbirth.^[13,21]^ As Hayakawa notes, humanity has developed new weapons against the infection based on microbiology, immunology and molecular biology. With these weapons, we were able to fight new enemies in the clinical arenas of obstetrics and infectious disease control with speed and precision that are incomparable to those of the early 20th century.^[23]^

Furthermore, we noted that during the year of the pandemic, none of the pregnant women tested positive for COVID-19 infection at the time of the stillbirth. However, it should be noted that there were SARS-CoV-2 infected pregnant women at the Department of gynecology and obstetrics at the hospital as well as outside of the hospital, included in this study, but none of those pregnancies ended in a stillbirth. This further emphasizes the qualitative care provided by University Hospital of Split to pregnant patients during the pandemic.^[21]^

Cause of perinatal death, classified with the ReCoDe classification system, showed no statistically significant difference between the studied groups. While the ReCoDe classification system is typically used to categorize stillbirths, in this study, it was utilized to classify all perinatal cases, including early neonatal deaths. We were able to allocate each case in ReCoDe category without significant data dispersion. Since classification systems used for perinatal death have many categories and subcategories, and are utilized for perinatal death statistics on a national level with a large study group and involving many cases, it would have been inappropriate to use them in our study due to the lower number of perinatal death cases. However, according to the national data, fetal category, more specifically the subcategory “intrauterine growth restriction” is the major cause of perinatal death. Our study contradicts this showing that category of “amniotic fluid” and its subcategory “chorioamnionitis” accounts for the majority of perinatal deaths with 18 (38%) and 15 (31%) the year before and the year during the COVID-19 pandemic, respectively. Moreover, the national data describes that when ReCoDe classification system is utilized, only 15% of all stillbirths are classified as unknown. Our study shows an even superior result with 2% being classified as unknown the year before the COVID-19 pandemic and 8% during the pandemic.^[14–16]^

Despite the aforementioned findings, there are some limitations of the study that should be acknowledged. We must note that our research shows the impact of the COVID-19 pandemic on adverse fetal outcomes, but not the impact of COVID-19 infection itself. Most of the pregnant women in our research had negative SARS-CoV-2 PCR tests at the time of admission to the hospital, which does not mean that they did not have a COVID-19 infection before that, which was not recorded in this study. Furthermore, our study included only 1 facility (i.e., the University Hospital of Split) and despite being a tertiary center covering a large area and despite the total number of 8446 births in 2 years, we consider it to be a relatively small sample size. Ideally, more facilities should have been included in this study.

## 5. Conclusion

No statistically significant difference in the incidence of unfavorable outcomes in pregnancy the year prior to the pandemic and the year during the COVID-19 pandemic at the University Hospital of Split was seen in this study. This can be interpreted as the pandemic did not have a negative effect on pregnant women and their fetuses. There was no increase in miscarriage, intrauterine fetal demise, or perinatal death during the year of the pandemic. Although our initial hypothesis was not confirmed, the study’s results do indicate that the University Hospital of Split was able to provide pregnant women with the same high-quality health care they required, despite the ongoing pandemic and additional restrictions that were introduced. All of which had no adverse effect on the final outcome of their pregnancies.

## Author contributions

**Conceptualization:** Andrea Dagelić, Emma Mulic, Ivana Kuzmic Prusac, Sandra Zekic Tomas.

**Data curation:** Andrea Dagelić, Emma Mulic, Ivana Kuzmic Prusac, Sandra Zekic Tomas.

**Formal analysis:** Andrea Dagelić, Emma Mulic, Ivana Kuzmic Prusac, Sandra Zekic Tomas.

**Investigation:** Andrea Dagelić, Emma Mulic, Ivana Kuzmic Prusac, Sandra Zekic Tomas.

**Methodology:** Andrea Dagelić, Emma Mulic, Sandra Zekic Tomas.

**Project administration:** Andrea Dagelić, Emma Mulic, Sandra Zekic Tomas.

**Resources:** Andrea Dagelić, Emma Mulic, Ivana Kuzmic Prusac, Sandra Zekic Tomas.

**Supervision:** Ivana Kuzmic Prusac, Sandra Zekic Tomas.

**Validation:** Ivana Kuzmic Prusac, Sandra Zekic Tomas.

**Writing – original draft:** Andrea Dagelić, Emma Mulic, Ivana Kuzmic Prusac.

**Writing – review & editing:** Andrea Dagelić, Emma Mulic, Sandra Zekic Tomas.
